# Pregnancy-shaped donor-directed alloimmune recall in husband-to-wife kidney transplantation: an integrative mechanistic framework

**DOI:** 10.3389/fimmu.2026.1936063

**Published:** 2026-09-03

**Authors:** Heng Zhang, Chaohua Feng, Zhiyuan Bai, Yajun Wang, Huaiyu Wang, Jiaxin Zhao, Weigang Wang

**Affiliations:** 1 Department of Urology II, The First Hospital of Jilin University, Changchun, China; 2 Department of Urology I, The First Hospital of Jilin University, Changchun, China

**Keywords:** antibody-mediated rejection, donor-specific antibody, husband-to-wife kidney transplantation, memory B cells, pregnancy sensitization

## Abstract

Spousal living donation expands access to kidney transplantation. Husband-to-wife kidney transplantation (HTW) may have a distinct immunologic context when the recipient has had pregnancies with the current donor, because maternal exposure involves the subset of paternal human leukocyte antigen (HLA) molecules and eplets inherited by the fetus rather than the donor’s complete HLA repertoire. We conducted a mechanism-focused narrative review using reproducible PubMed/MEDLINE searches from database inception through 3 July 2026, supplemented by targeted checks of Embase and the Web of Science Core Collection, reference-list screening, and forward and backward citation tracking. Contemporary cohorts generally report acceptable aggregate patient and graft outcomes after modern immunologic screening. However, early donor-specific antibody (DSA) increases, microvascular inflammation (MVI), and antibody-mediated rejection (ABMR) are concentrated among recipients with high panel-reactive antibody or calculated panel-reactive antibody, current or historical DSA, positive flow cytometry crossmatch, or low-level or missed preformed DSA. We propose a hypothesis-generating, two-branch framework. Branch A comprises persistence or rebound of low-level or missed preformed DSA, whereas Branch B comprises reactivation of donor-reactive memory B cells with an anamnestic response; the branches may coexist and converge on DSA-associated endothelial injury, MVI, and ABMR. DSA first detected after transplantation may reflect previously unrecognized preformed antibody, rebound or re-emergence of historical DSA, a memory-mediated anamnestic response, or a primary response to the graft; timing alone cannot distinguish these possibilities. Evidence remains incomplete because no prospective HTW cohort has linked pregnancy-relevant paternal antigen exposure, pretransplant donor-reactive memory B cells, same-specificity posttransplant DSA, and contemporaneous Banff-classified biopsy findings. Shared pregnancy should be treated as a possible donor-related exposure, not a universal risk label; current evidence does not support excluding a husband donor or changing treatment solely on this basis.

## 1 Introduction

Living-donor kidney transplantation can shorten waiting times for patients with kidney failure and remains an important source of transplantable kidneys. Amid persistent organ shortages, spousal donation offers a practical living unrelated donor option for candidates without a suitable related donor (1, 2). Although spouses are usually genetically unrelated, this approach provides an available donor, enables planned surgery, and builds on an established caregiving relationship (3). Husband-to-wife kidney transplantation (HTW), however, is not always immunologically equivalent to other living unrelated donor kidney transplants. When a couple has shared pregnancies, the wife may previously have encountered paternal human leukocyte antigen (HLA) molecules and related molecular epitopes inherited and expressed by a shared fetus (4). Thus, HTW describes a donor relationship and, in selected couples, may also identify a donor-related antigen-exposure context requiring more specific immunologic interpretation.

The defining feature is not marriage itself but whether a shared pregnancy created paternal antigen exposure relevant to the current graft. A fetus generally inherits and expresses one paternal HLA haplotype. The mother therefore encounters the paternal HLA molecules and eplets expressed by that fetus, not the husband’s complete HLA repertoire (4, 5). Marriage alone does not establish immunologic re-exposure. Transplantation from the husband constitutes a more direct antigen re-encounter only when the graft expresses paternal HLA/eplets previously displayed by a shared fetus. Cross-reactive eplets overlapping that exposure may also be relevant (6–8). Evidence of shared pregnancy, biological paternity, offspring HLA/eplets, and historical serum samples strengthens assessment of whether the graft corresponds to prior paternal antigen exposure.

The clinical question arises from an apparent discrepancy between early humoral signals and contemporary overall outcomes. In earlier testing eras, adverse events occurred even after negative complement-dependent cytotoxicity crossmatch (CDC-XM) or flow cytometry crossmatch (FCXM) results. Case reports, small series, and retrospective single-antigen bead analyses documented rapid posttransplant changes in donor-specific antibody (DSA), microvascular inflammation (MVI), and C4d-positive or C4d-negative antibody-mediated rejection (ABMR). Rejection-related graft loss was also reported in some HTW recipients and, more broadly, in some female recipients of spousal kidneys (7–10). In this review, DSA is used in its standard sense: an antibody directed against one or more HLA antigens expressed by the current donor. The term does not identify the sensitizing event; pregnancy attribution is assessed separately. In modern cohorts using single-antigen bead assay (SAB), virtual crossmatch, desensitization, induction therapy, and contemporary maintenance immunosuppression, HTW has not consistently been associated with worse long-term patient or graft survival (11, 12). Risk-stratified studies instead suggest that excess events cluster among recipients with established humoral risk. Relevant features include high panel-reactive antibody (PRA) or calculated PRA (cPRA), current pretransplant DSA, historical DSA, positive FCXM, or low-level or missed preformed DSA (12–14).

Earlier interpretations often treated pregnancy as a static sensitizing event and relied on parity, PRA/cPRA, or currently detectable anti-HLA antibodies. They less often distinguished antibody waning, historical DSA, low-level preformed DSA, and latent immunologic memory (15, 16). A single pretransplant SAB result may also be overinterpreted because assays, thresholds, and interference differ, and older CDC-XM or FCXM results are not equivalent to modern SAB testing. Thus, “not currently detected” may be mistaken for “never immunologically imprinted” (17, 18). Similarly, DSA first detected after transplantation is often labeled *de novo* DSA without adequately separating preformed or missed low-level antibody, reactivation of prior immune memory, and a primary response to the graft (10, 18). Many studies also lack confirmation of shared pregnancy and biological paternity, offspring HLA/eplet mapping, historical sera, and donor HLA-reactive memory B-cell data. These omissions limit pregnancy-source attribution and assessment of whether such variables add information beyond cPRA, current pretransplant DSA, historical DSA, and crossmatch results (7, 19).

Previous reviews have generally addressed pregnancy-related HLA sensitization, maternal–fetal tolerance, memory B-cell biology, or HTW outcomes separately (5, 7, 15, 16). Few have integrated shared-pregnancy antigen exposure, latent donor reactivity, early DSA kinetics, MVI/ABMR, and possible incremental value beyond routine measures within one framework (10, 20, 21). Here, static “pregnancy sensitization” is reframed as a dynamic process of donor-directed humoral recall. The framework links paternal HLA/eplets expressed by a shared fetus to antigens on the husband’s graft. It distinguishes current pretransplant DSA, historical DSA, low-level or missed preformed DSA, persistent antibody maintained by long-lived plasma cells, and memory B-cell–mediated recall, while focusing on early DSA kinetics, MVI, and C4d-positive or C4d-negative ABMR. **Supplementary Table S1** summarizes established terminology and interpretive cautions relevant to this review. This explanatory framework does not classify all HTW procedures as high risk, redefine DSA, or establish a ready-to-use clinical decision rule. No new diagnostic categories or consensus terminology are proposed.

## 2 Review methods

This mechanism-focused narrative review was based on a structured literature search (22, 23). PubMed/MEDLINE was selected as the primary database because the review question was biomedical and clinically focused; it provided the reproducible Boolean strategies and retrieval counts reported here. To broaden coverage, we also conducted targeted checks of Embase and the Web of Science Core Collection, screened reference lists, and performed forward and backward citation tracking. These supplementary checks were not treated as independent structured database searches; database-specific strategies and retrieval counts were therefore not reported. PubMed/MEDLINE was searched from inception through 3 July 2026. Search concepts covered husband-to-wife or spousal kidney transplantation, pregnancy-associated HLA alloimmunization, donor-specific humoral memory, donor-specific antibody, microvascular inflammation, and antibody-mediated rejection. Complete PubMed strategies, field tags, limits, the search date, retrieval counts, and the role of the supplementary database checks are provided in **Supplementary Table S2**.

Eligible evidence comprised four sources: direct HTW clinical studies; female-spousal transplantation studies in which shared pregnancy or biological paternity was incompletely established; pregnancy-immunology and antigen-attribution studies; and general mechanistic studies in transplantation that directly informed an HTW-specific link in the proposed framework. Reports without relevant clinical outcomes or direct mechanistic relevance were excluded. References were imported into Zotero (version 9.0.6; Corporation for Digital Scholarship, Vienna, VA, USA) for organization and duplicate detection, and title/abstract and full-text screening decisions were recorded in Microsoft Excel 365 (Microsoft Corporation, Redmond, WA, USA). The six PubMed searches yielded 920 records before deduplication across searches; 680 unique PubMed records remained after removal of 240 duplicates and were screened by title and abstract. Candidate full texts were assessed against the eligibility criteria, and 85 publications were included in the narrative synthesis, including the 12 clinical studies summarized in **Table 1**; eligibility uncertainties were resolved by discussion within the review team.

**Table 1:** Clinical studies informing husband-to-wife kidney transplantation and pregnancy-source interpretation.

Study	Design and population	Pregnancy/antigen-source information	Immunologic profile	Endpoint(s) and timing	Main finding	Clinical directness | pregnancy-source attribution	Main interpretive limitation
Group 1. Early clinical signals after negative or limited-sensitivity screening							
Rosenberg et al. (2004) (8)	Two direct HTW cases: one accelerated-rejection case and one positive-FCXM transplant after plasmapheresis.	Case 1: G5P5 and no transfusion. Case 2: G3P3 plus transfusion. Biological paternity and offspring HLA/eplet data were unavailable.	Case 1: PRA, cytotoxic XM, and FCXM negative; POD3 donor-lymphocyte antibody test negative. Case 2: PRA 17%, anti-HLA-A3, positive FCXM converted to negative after plasmapheresis.	Case 1: POD2 Banff IIA rejection and 2-year follow-up. Case 2: 4-month graft function; recurrent disease by 3 years. C4d was unavailable.	Case 1 sCr peaked at 4.3 mg/dL on POD6 and was 1.2 mg/dL at 2 years; case 2 sCr was 1.2 mg/dL at 4 months.	Direct HTW evidence | limited pregnancy-source attribution	The principal accelerated event was predominantly cellular without detected circulating donor antibody; the second case was confounded by transfusion and desensitization.
Ghafari (2008) (27)	Single-center experience among 1,350 transplants; multiparous subgroups included offspring-to-mother (n = 12), HTW (n = 9), and other living-unrelated grafts (n = 150).	All recipients had 2–5 children; shared paternity, mutual-child status, and offspring HLA/eplets were not verified.	AHG-CDC XM negative in all cases; PRA details were unclear, and DSA/SAB/FCXM were NR.	Hyperacute graft loss, acute rejection per patient-year, and 1-, 3-, and 5-year graft survival.	HTW hyperacute graft loss occurred in 2/9 (22.2%); graft survival was 78%, 75.2%, and 68.5% at 1, 3, and 5 years; acute rejection was 0.81 episodes/patient-year.	Direct HTW evidence | limited pregnancy-source attribution	Very small HTW subgroup and older testing era; source table percentages were internally inconsistent, so textual results were used cautiously.
Matsuo et al. (2009) (9)	Direct HTW case: 59-year-old wife recipient and 58-year-old husband donor; ABO-compatible, 5/6 HLA-A/B/DR mismatch.	Two pregnancies with the donor; transfusion explicitly absent; offspring HLA/eplet data unavailable.	Warm T- and B-cell CDC-XM results were negative; cold B-cell CDC-XM was weakly positive, FCXM and flow PRA negative; retrospective LABScreen SAB detected weak pretransplant B35 DSA.	POD3 rejection; biopsies on POD7 and POD34; 6-month follow-up.	POD7 acute vascular rejection/acute ABMR type II (g2–3, v1–2, ptc3, C4d-positive); sCr 0.9 mg/dL by POD22; antibody negative on POD90; graft functioning at 6 months.	Direct HTW evidence | moderate pregnancy-source attribution	Single case; no offspring antigen mapping or memory B-cell assay, and the weak preformed DSA was demonstrated only retrospectively.
Groeneweg et al. (2021) (10)	Single-center cohort of 352 ABO-compatible, CDC-XM-negative living-unrelated transplants (1997–2014): female recipients of male spousal donors, n = 61; female recipients of male nonspousal donors, n = 36; recipients of female donors, n = 46; and male recipients, n = 209.	Pregnancy/transfusion data were incomplete; pregnancy with the donor’s child was reported in 4/6 early-ABMR cases and 23/35 without ABMR among those with data.	Retrospective PAB/SAB testing identified preformed DSA in 7/9 early-ABMR cases (median MFI 2,200), including 5/6 female-spousal cases; PAB was positive in 3/9 and C4d in 8/9.	Histologic rejection within 6 months; early ABMR defined as ≤14 days; 1-year graft loss/survival.	Early ABMR: 6/61 (10%) female-spousal recipients vs. 0/36, 2/46, and 1/209 comparators; RR 9.5, p < 0.001; median 8 days. Four of 9 early-ABMR grafts were lost; 1-year death-censored survival was 56%.	Female-spousal evidence | limited pregnancy-source attribution	Shared pregnancy was incompletely established; early ABMR was often explained by missed preformed DSA rather than proven memory B-cell recall.
Group 2. Modern general or standard-risk spousal/HTW cohorts							
Guad et al. (2019) (29)	Malaysian single-center cohort of first grafts (1996–2014): spousal donors n = 32 (8 female recipients/HTW, 24 male recipients/WTH) vs. living-related donors n = 109.	Pregnancy, mutual-child, paternity, and offspring HLA/eplet information were unavailable.	ABO-compatible and T-cell lymphocytotoxic XM negative; two HLA-A mismatches, 26% vs. 3% (p = 0.0004) and HLA-B ≥1 mismatch 90% vs. 70% (p = 0.039); DSA/PRA were NR.	Acute rejection, creatinine, and graft survival at 6 months, 1 year, and 3 years.	Spousal graft survival was 100%, 91%, and 88% at 6 months, 1 year, and 3 years; acute rejection was 22%, 25%, and 38%. At 1 year, 3 HTW and 6 WTH rejection episodes were reported.	Direct HTW evidence | limited pregnancy-source attribution	The HTW subgroup was incompletely tabulated; DSA status and rejection subtype (ABMR vs cellular) were unavailable.
Kim et al. (2020) (11)	Korean single-center retrospective cohort of 390 parous female recipients: offspring-to-mother n = 175, HTW n = 159, other unrelated donors n = 56; HLA-incompatible, retransplant, and CDC/FCXM-positive procedures excluded.	Parity was required, but shared pregnancy, biological paternity, offspring HLA/eplets, and historical sera were unavailable.	HTW: pretransplant DSA 45/159, ABO-incompatible 54/159, HLA-A/B/DR mismatch 4.4 ± 1.2; positive CDC-XM/FCXM excluded.	BPAR, clinical rejection, posttransplant first-detected DSA, DGF, mortality, and 5-year graft outcomes.	HTW BPAR 11.3%; 5-year BPAR-free survival 88.7%, graft survival 95.5%, and death-censored graft survival 97.5%. Pretransplant DSA independently predicted BPAR (adjusted HR 2.10, 95% CI 1.06–4.15).	Direct HTW evidence | limited pregnancy-source attribution	Modern selection excluded higher-risk combinations; pregnancy-source continuity, early serial DSA, and contemporaneous biopsy findings were unavailable.
Luo et al. (2025) (28)	Chinese single-center spousal cohort (2007–2021): HTW n = 33 vs. WTH n = 131; all crossmatch-negative and without retransplantation.	All female recipients were parous; HTW pregnancy count 2 ± 1; shared paternity and offspring HLA/eplets were unavailable.	Pretransplant DSA, CDC-XM, and FCXM negative; HTW HLA mismatch 4.2 ± 1.4; ABO-incompatible 1/33.	For-cause biopsy after ≥20% creatinine rise; Banff 2017; 2-year BPAR, ABMR/TCMR, posttransplant first-detected DSA, function, and survival.	Two-year BPAR was 6/33 vs. 7/131 (p = 0.03); HTW events comprised 5 TCMR and 1 ABMR. One HTW recipient developed posttransplant first-detected DSA at 1 week with graft loss; patient/graft survival was similar.	Direct HTW evidence | limited pregnancy-source attribution	Small HTW group and TCMR-dominant endpoint; offspring antigen data and memory B-cell testing were unavailable.
Group 3. Shared-pregnancy-informed or immunologically enriched HTW evidence							
Senn et al. (2021) (7)	Basel single-center cohort (2005–2020) of previously pregnant women receiving a first HLA-mismatched graft: HTW n = 25, other living donors n = 52, deceased donors n = 120.	Shared children required; donor and recipient were interviewed separately about biological paternity. HTW pregnancies: 1 in 16%, 2 in 52%, ≥3 in 32%; offspring HLA/eplets unavailable.	SAB Luminex cutoff 500 MFI; virtual-crossmatch DSA; HTW pretransplant DSA 9/25, median cPRA 1%; T/B-cell CDC-XM negative.	Protocol biopsies at 3 and 6 months plus for-cause biopsy; Banff 2015; median follow-up 5.1 years; median timing of postoperative DSA testing, 3.1 years.	Death-censored graft survival differed (p = 0.03); 3/4 HTW graft failures were rejection-related; 5-year patient survival 100%. ABMR/mixed rejection 36% vs. 20% vs. 18% (p = 0.10); 1-year clinical rejection 28% vs. 22% vs. 15% (p = 0.19).	Direct HTW evidence | moderate pregnancy-source attribution	Small HTW sample; no offspring antigen mapping or memory B-cell testing, and DSA/biopsy sampling was asynchronous.
Drissi Bourhanbour et al. (2021) (30)	Two spousal case reports; one direct HTW case: a 33-year-old woman received a kidney from her husband.	HTW recipient had one pregnancy (2008) and a transfusion; offspring HLA/eplets and formal paternity verification were unavailable.	One Lambda SAB positive threshold 1,000 MFI; pretransplant screening was reported as negative, but retrospective analysis demonstrated five low-level DSA (A26, B52, DR1, DR15, DQ6); CDC-XM negative.	Pretransplant and POD30 DSA kinetics; 1-year graft function; no biopsy-proven rejection endpoint.	At POD30, B52 rose 389.65→3,390.57, DR15 139.41→3,381.87, and DQ6 505.99→5,380.93 MFI; anti-A11 appeared at an MFI of 637.12. After tacrolimus intensification, the graft functioned at 1 year and anti-HLA screening was negative.	Direct HTW evidence | limited pregnancy-source attribution	Single HTW case without biopsy; transfusion was a competing sensitizing event, and pregnancy-source attribution could not be isolated.
Tanabe et al. (2025) (14)	Clinicopathologic series of 7 FCXM-positive, highly sensitized recipients given H-IVIG-based desensitization; 4 were direct HTW cases.	Pregnancy was reported for HTW cases 2, 3, and 7; case 4 also had transfusion. Offspring HLA/eplets and paternity confirmation were unavailable.	B-cell FCXM was positive in all seven recipients before and after desensitization; T-cell FCXM 6/7 before and 3/7 after. SAB threshold > 1,000 MFI; HTW maximum DSA MFI before/at transplant: 1,400/1,221; 6,547/2,241; 735/1,411; 246/68.	Acute rejection within 1 month; serial protocol biopsies through 36 months; mean follow-up 36 months.	Three of 7 had acute rejection within 1 month. Case 3 had day-6 acute ABMR (g3, ptc1, v1, C4d2); cases 2 and 4 had early TCMR followed by chronic active ABMR/mixed rejection. All grafts functioned at follow-up.	Direct HTW evidence | moderate pregnancy-source attribution	Small, desensitized FCXM-positive series not representative of standard-risk HTW; competing sensitization and intervention confound attribution.
Choi et al. (2026) (12)	Retrospective cohort of 455 spousal transplants: standard-risk PRA < 50% (WTH n = 291, HTW n = 75) and high-risk PRA ≥50% (WTH n = 34, HTW n = 55).	Pregnancy was recognized as a sensitizing source, but detailed histories were available for only 43 patients and were not analyzed; offspring HLA/eplets were unavailable.	CDC-XM, FCXM, and SAB-based DSA were assessed. High-risk B-cell FCXM positivity: HTW 23/55 (41.8%) vs. WTH 4/34 (11.8%), p < 0.001.	One-year BPAR with ABMR/TCMR, posttransplant first-detected DSA, eGFR, infections, and up to 10-year patient/graft survival.	Standard-risk 1-year BPAR: WTH 8.0% vs. HTW 5.7% (p = 0.10). High-risk: HTW 20.4% vs. WTH 3.1% (p = 0.03), all acute ABMR; adjusted HR 2.35 (95% CI 1.11–4.98). Long-term survival did not differ.	Direct HTW evidence | limited pregnancy-source attribution	Strong risk-concentration signal, but detailed pregnancy attribution and donor-reactive memory assays were unavailable.
Ozkara et al. (2025) (13)	Hacettepe single-center DSA-positive cohort (2010–2020): HTW n = 20, WTH n = 39, living-related donors n = 72; HTW follow-up 36 ± 16.3 months.	Shared children and pregnancy history were reported only at the group level; offspring HLA/eplets and individual paternity mapping were unavailable.	Luminex DSA threshold 750 MFI; HTW class I DSA 15/20 and class II 13/20; ABO-compatible, T/B lymphocytotoxic XM and FCXM negative; no desensitization.	One-year acute rejection/subtype, 5-year rejection risk, creatinine doubling, and graft loss.	One-year rejection: HTW 6/20 (30%), WTH 6/39 (15%), related 7/72 (10%), p = 0.01; HTW vs. related OR 4.231 and HR 3.734. The 5-year HR of 1.203 was not statistically significant; no HTW graft loss was reported.	Direct HTW evidence | moderate pregnancy-source attribution	Small retrospective cohort; postoperative DSA kinetics, protocol biopsy, C4d/MVI, and offspring antigen mapping were unavailable.

Groups reflect each study’s main role in the review, not publication year. Clinical directness and pregnancy-source attribution are descriptive judgments. Attribution is stronger when shared pregnancy, biological paternity, child HLA/eplet data, historical sera, or temporal antibody continuity are available; missing data do not exclude pregnancy-related sensitization but limit source attribution. These narrative assessments should not be interpreted as formal certainty-of-evidence ratings. NR indicates not reported. “Absent” is used only when the original study explicitly confirmed absence; otherwise, “not reported,” “not assessed,” or “unavailable” is used. Effect estimates are presented only when reported by the original study. Unless an original study specified a different cutoff, “early” denotes events occurring within 30 days after transplantation; first-year outcomes are reported separately and are not categorized as early events. Posttransplant first-detected DSA refers to DSA first identified after transplantation using the pretransplant assays and thresholds available in the original study; the designation does not establish a primary immune response.

HTW, husband-to-wife kidney transplantation; WTH, wife-to-husband kidney transplantation; LDKT, living-donor kidney transplantation; LURKT, living unrelated kidney transplantation; HLA, human leukocyte antigen; ABO, ABO blood group system; XM, crossmatch; AHG-CDC XM, antihuman globulin-enhanced complement-dependent cytotoxicity crossmatch; DSA, donor-specific antibody against current donor HLA; CDC-XM, complement-dependent cytotoxicity crossmatch; FCXM, flow cytometry crossmatch; SAB, single-antigen bead assay; PAB, panel antigen bead; PRA, panel-reactive antibody; BPAR, biopsy-proven acute rejection; TCMR, T cell-mediated rejection; ABMR, antibody-mediated rejection; MVI, microvascular inflammation; C4d, complement split product C4d; H-IVIG, high-dose intravenous immunoglobulin; eGFR, estimated glomerular filtration rate; DGF, delayed graft function; G, gravida; P, para; POD, postoperative day; ATG, antithymocyte globulin; CI, confidence interval; HR, hazard ratio; MFI, mean fluorescence intensity; MP, methylprednisolone; OR, odds ratio; PEX, plasma exchange; RR, relative risk; sCr, serum creatinine.

Evidence was synthesized descriptively rather than quantitatively. Direct HTW studies and female-spousal transplantation studies with incomplete documentation of shared pregnancy or biological paternity informed the clinical phenotype relevant to HTW (7). Pregnancy-immunology studies informed paternal-antigen exposure and attribution (24), and general transplant studies were retained only when they clarified preexisting antibody, memory-mediated recall, or DSA-associated graft injury in the HTW context (16, 26). HLA antibody testing, DSA, memory responses, and alloimmune assays were treated as explanatory variables rather than scored or combined into an evidence grade (18). No meta-analysis, formal risk-of-bias assessment, or certainty-of-evidence grading was performed.

## 3 Clinical and epidemiologic evidence

Clinical studies of HTW differ substantially in testing era, immunologic selection, completeness of pregnancy information, and endpoint definition. Direct HTW studies were distinguished from female-spousal transplantation studies; pregnancy-immunology and general mechanistic evidence from transplantation are used primarily in Section 4 and **Table 2**. **Table 1** summarizes study design, population, pregnancy or antigen-source information, immunologic profile, clinical endpoints, principal findings, and interpretive limitations. The following sections synthesize findings from earlier, less sensitive testing eras and outcomes under modern screening, examine studies with stronger antigen attribution or enriched immunologic risk, and compare MVI/ABMR, biopsy-proven acute rejection (BPAR), DSA kinetics, and long-term graft outcomes. Because timing definitions varied across studies, study-specific timing is reported in **Table 1**. In this review, “early” is used descriptively for events occurring within 30 days after transplantation unless an original study specified another cutoff; outcomes assessed over the first year are described as first-year outcomes rather than early events.

**Table 2:** Mechanistic nodes, convergent evidence, and unresolved links in pregnancy-shaped donor-directed humoral recall.

Mechanistic node/link	HTW or female-spousal clinical signal	Cross-source mechanistic support	Integrated interpretation	Key unverified link
Shared pregnancy and inherited paternal HLA/eplet exposure	HTW studies that documented shared pregnancy or assessed biological paternity support a plausible donor-related re-exposure setting (7, 9).	Child-specific HLA/eplet and pregnancy antigen-attribution studies (6, 15, 24, 25, 34, 35).	Biologically plausible upstream exposure, but not proof of donor-directed sensitization.	Mother–child–donor HLA/eplet overlap, historical sera, and competing sensitization sources traced in the same HTW cohort.
Antibody waning and latent donor-directed humoral reservoir	Early events after negative conventional testing; retrospective or low-level preformed DSA in HTW/female-spousal reports (9, 10, 30).	Antibody waning; LLPCs; HLA-reactive memory B cells; rebound kinetics (16, 37, 42–45, 50).	Seronegativity may conceal prior sensitization or latent humoral memory.	Historical DSA specificity and pretransplant donor-reactive memory B cells linked to the current donor.
Branch A: low-level or missed preformed DSA persistence/rebound	Early DSA increase, MVI, or ABMR after negative or limited-sensitivity testing (9, 10, 30).	Low-level preformed DSA; retrospective SAB; dilution, prozone, and threshold effects (39–41, 58).	Some DSA first detected after transplantation may represent persistence or rebound of previously missed preformed DSA.	Same-specificity low-level or historical pretransplant DSA demonstrated by sensitive assays, dilution studies, and historical-serum analysis.
Branch B: memory B-cell–mediated anamnestic DSA response	Rapid posttransplant DSA rise or early ABMR is consistent with recall but does not directly demonstrate a memory B-cell origin (9, 10, 12, 28).	Pregnancy-related HLA-reactive memory B cells; plasmablast differentiation; anamnestic kinetics (16, 42–45, 58).	Rapid timing is compatible with recall but cannot by itself distinguish an anamnestic response from missed preformed DSA.	Missed preformed DSA excluded, with pretransplant donor HLA-reactive memory B cells linked to same-specificity posttransplant DSA.
DSA-mediated endothelial injury, MVI, and ABMR	DSA with MVI, C4d, or ABMR in HTW/female-spousal reports; humoral phenotypes in high-risk HTW (9, 10, 12–14).	Banff ABMR framework; complement- and Fc receptor–mediated endothelial injury (56, 61–67, 70).	The downstream DSA→MVI/ABMR link is comparatively mature; pregnancy source often remains unresolved.	Pregnancy-linked antigen overlap, same-specificity DSA, contemporaneous biopsy phenotype, and longitudinal graft outcomes traced in the same HTW cohort.

This table summarizes mechanistic nodes rather than individual studies. Clinical signals refer to direct HTW studies or closely related female-spousal donor settings. Cross-source support includes pregnancy alloimmunization, child-specific HLA/eplet and pregnancy antigen-attribution studies, memory B-cell and plasma-cell biology, DSA kinetics, and ABMR/MVI literature. Branches A and B may coexist and cannot be distinguished by posttransplant timing alone. The table neither assigns evidence scores nor establishes a continuous causal chain.

HTW, husband-to-wife kidney transplantation; HLA, human leukocyte antigen; DSA, donor-specific antibody against current donor HLA; SAB, single-antigen bead assay; LLPC, long-lived plasma cell; MVI, microvascular inflammation; ABMR, antibody-mediated rejection; C4d, complement split product C4d; Fc, fragment crystallizable.

### 3.1 Early immunologic signals in the era of limited-sensitivity testing

Case reports and small cohorts from earlier, less sensitive testing eras documented early rejection after apparently negative conventional screening. Rosenberg et al. described accelerated rejection (8), Ghafari reported hyperacute graft loss (27), Matsuo et al. retrospectively identified weak preformed DSA in a shared-pregnancy HTW case with early C4d-positive ABMR and MVI (9), and Groeneweg et al. identified preformed DSA missed by conventional screening in female-spousal recipients with early ABMR (10). Taken together, these studies indicate that a negative conventional screen did not exclude low-level or otherwise undetected preformed DSA; the first days to weeks after transplantation therefore represented an important window in which a preexisting humoral background could become clinically apparent. Study-specific quantitative findings, assay details, and pathologic assessments are summarized in **Table 1**.

### 3.2 Overall cohorts under modern screening

Modern screening-era cohorts generally reported acceptable outcomes after appropriate recipient selection. Kim et al. showed no worse aggregate HTW outcomes after HLA-incompatible and crossmatch-positive procedures were excluded (11). The higher BPAR rate reported by Luo et al. was driven mainly by TCMR and therefore did not specifically support a humoral mechanism (28). Guad et al. likewise reported acceptable spousal-transplant outcomes, but shared-pregnancy, paternity, offspring-antigen, and rejection-subtype data were limited or unavailable (29). These cohorts do not exclude risk within immunologically defined HTW subgroups.

### 3.3 Studies with stronger antigen attribution or enriched immunologic risk

Senn et al. and Matsuo et al. provide comparatively strong evidence in a shared-pregnancy context, although child-specific antigen overlap and longitudinal continuity from historical sensitization to the posttransplant response were not established (7, 9).

Choi et al., Ozkara et al., Tanabe et al., and Drissi Bourhanbour et al. collectively suggest that clinically relevant events are concentrated among recipients with high PRA/cPRA, current DSA, a positive FCXM, or low-level or missed preformed DSA (12–14, 30). These findings support risk stratification by established humoral features rather than classification of all husband donors as high risk.

### 3.4 Cross-study synthesis by clinical endpoint

ABMR and MVI are the endpoints most directly aligned with the proposed humoral axis (7, 9, 10, 12, 14), whereas unclassified BPAR and TCMR provide only supportive or nonspecific signals (11, 28, 29). DSA first detected after transplantation may represent previously unrecognized preformed antibody, rebound or re-emergence of historical DSA, a memory-mediated anamnestic response, or a primary response to the graft (9, 10, 13, 30). Timing alone cannot distinguish these possibilities. Long-term outcome differences remain inconsistent or attenuated across studies (7, 11–13, 27–29).

### 3.5 Early humoral risk signals and attenuated or inconsistent long-term differences

Current evidence does not support HTW as a homogeneous high-risk category. Aggregate BPAR, patient survival, and graft survival are generally acceptable in cohorts selected with contemporary methods (11, 28). Early DSA increases and MVI/ABMR may cluster in specific settings, whereas some studies also report increased first-year BPAR. Such settings include negative conventional testing followed by retrospective identification of missed preformed DSA, comparatively strong shared-pregnancy documentation, and high PRA, current DSA, or positive FCXM (7–10, 12, 13, 27, 30). Early signals and weaker long-term differences are not contradictory. Donor selection and paired exchange may exclude the highest-risk combinations, while virtual crossmatch, treatment, and surveillance may interrupt progression; small samples and few events also reduce power to detect long-term differences. The phenotype requiring mechanistic explanation is therefore early DSA rebound, MVI, and ABMR in immunologically enriched HTW recipients.

## 4 Core mechanistic pathway

**Figure 1:**
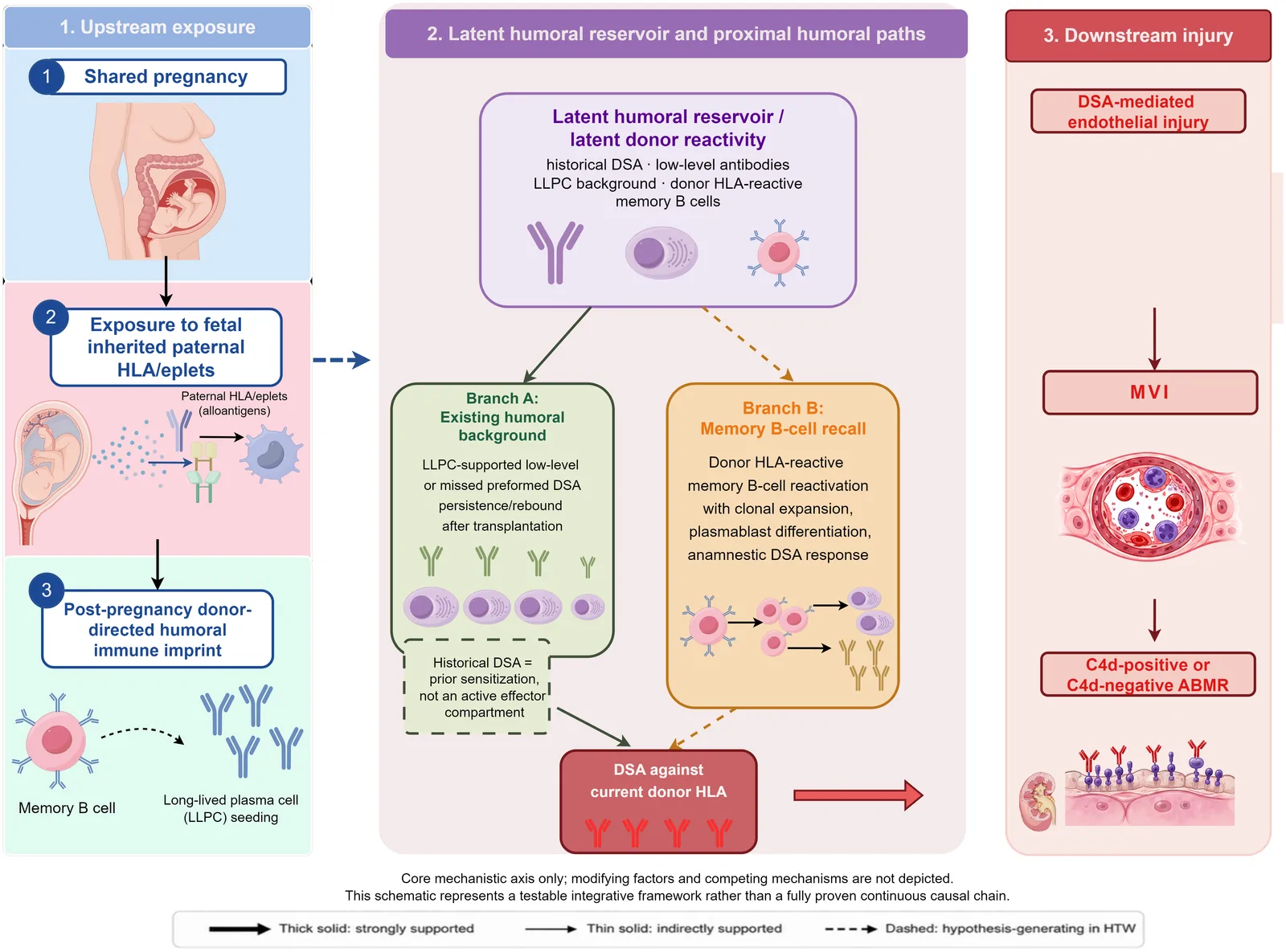
Core mechanistic axis of pregnancy-shaped donor-directed humoral recall in husband-to-wife kidney transplantation. Shared pregnancy may establish a donor-directed humoral imprint against paternal HLA/eplets inherited by the fetus. After transplantation, this latent reservoir may become clinically manifest through two distinct, potentially coexisting pathways: Branch A, persistence or rebound of low-level or missed preformed DSA, potentially supported by long-lived plasma cells; and Branch B, reactivation of donor-reactive memory B cells with plasmablast differentiation and an anamnestic DSA response. Both may converge on endothelial injury, MVI, and C4d-positive or C4d-negative ABMR. Historical DSA indicates prior sensitization rather than an effector compartment. Arrow styles indicate evidentiary strength. Branches A and B may coexist, and early posttransplant timing alone cannot distinguish persistence or rebound of preformed DSA from memory B-cell-mediated recall. The schematic is a hypothesis-generating framework rather than a proven continuous causal chain. Endothelial injury, MVI, and ABMR are presented as related pathologic manifestations rather than obligatory sequential temporal stages.

Section 3 defines an early humoral phenotype in which rapid DSA changes, MVI, and ABMR cluster among HTW recipients with higher immunologic risk. This section integrates HTW observations with human pregnancy alloimmunization, paternal HLA/eplet exposure, and established mechanisms of DSA-mediated graft injury. We propose a hypothesis-generating framework (**Figure 1**) that can be tested prospectively by integrating paternal HLA/eplets inherited by shared offspring, historical serum findings, pretransplant donor-reactive memory B-cell assays, serial same-specificity posttransplant DSA, and contemporaneous graft histology. It should not be interpreted as a proven continuous causal sequence. It comprises a shared upstream exposure, two distinct proximal humoral branches, and a common downstream injury pathway. Shared pregnancy may expose the recipient to paternal HLA/eplets inherited and expressed by the fetus, leaving a postpregnancy donor-directed humoral immune imprint. After transplantation, this latent state may become clinically manifest through Branch A, Branch B, or both. The evidentiary strength differs between the two proximal branches: Branch A has more direct clinical support from prospectively known or retrospectively demonstrated preformed DSA and same-specificity rebound, whereas Branch B remains predominantly mechanistic and inferential in HTW. Branch A reflects a preexisting humoral background that may be maintained by long-lived plasma cells and becomes evident as persistence or rebound of low-level or missed preformed DSA. Historical DSA documents prior sensitization but is not an effector compartment. Branch B involves reactivation of donor HLA-reactive memory B cells, followed by clonal expansion, plasmablast differentiation, and an anamnestic DSA response. Both branches may converge on DSA against current donor HLA, endothelial injury, MVI, and C4d-positive or C4d-negative ABMR.

### 4.1 Immune initiation: paternal HLA and molecular-epitope exposure during shared pregnancy

Shared pregnancy can constitute an upstream antigen exposure related to the current husband donor. Because a fetus inherits only one paternal HLA haplotype, the mother encounters paternal HLA molecules and eplets inherited by the fetus rather than the husband’s complete HLA repertoire (4, 6, 15, 24, 25, 31). Different shared offspring may broaden the set of paternal antigens encountered, and offspring HLA/eplet data can strengthen assessment of whether the graft re-expresses pregnancy-relevant targets (7, 15, 32, 33). The number and outcome of pregnancies, obstetric events, and time since the last pregnancy may modify exposure, but none of these factors has been validated as an HTW predictor.

Eplet analysis may refine antibody-relevant overlap between paternal HLA/eplets inherited by shared offspring and the husband’s graft. Predicted indirectly recognizable HLA epitopes presented by HLA class II (PIRCHE-II) may estimate the potential for indirect T-cell help; neither measure has been validated as a clinical threshold in HTW (25, 34, 35).

### 4.2 Local maternal–fetal tolerance and systemic humoral sensitization can coexist

Maternal–fetal tolerance is predominantly maintained within the placental–decidual environment and does not imply systemic tolerance to all paternal HLA. Pregnancy-related tolerance and humoral sensitization may therefore coexist. This coexistence becomes relevant when a kidney graft from the husband re-expresses overlapping paternal antigens in the inflammatory milieu of transplantation (15, 16, 33, 36).

### 4.3 Immune latency: donor reactivity after serum antibody waning

A negative result on current SAB testing does not establish that donor-directed sensitization never occurred. Results obtained with older PRA assays, CDC-XM, and FCXM are not equivalent to contemporary SAB findings, and historical DSA is a record of prior sensitization rather than an effector compartment (37–41). Retrospective identification of low-level or missed preformed DSA after negative conventional testing supports Branch A (9, 10). Antibody persistence supported by long-lived plasma cells and memory B-cell–mediated recall are biologically distinct. Evidence from the broader plasma-cell literature supports the plausibility of persistence but is not direct HTW evidence (50–55). Long-lived plasma cells may maintain antibody production without renewed antigen stimulation, whereas memory B cells require antigen re-encounter before clonal expansion and differentiation into antibody-secreting cells.

Individuals without detectable serum DSA may retain donor-reactive memory B cells. Several research assays can detect HLA- or donor-reactive memory B cells, but none is validated for routine HTW risk assessment (16, 42–49). Importantly, no prospective HTW study has linked pretransplant donor-reactive memory B cells to same-specificity posttransplant DSA and contemporaneous biopsy-proven ABMR.

### 4.4 Immune recall: antigen re-encounter after transplantation from the husband donor

The latent states described above become clinically relevant when the husband’s graft re-expresses overlapping paternal HLA/eplets. Pregnancy-associated exposure occurs in the placental–decidual environment, whereas transplantation presents these targets on directly accessible graft endothelium in an inflammatory setting (15, 36, 56, 57). Latent donor-directed humoral memory may then become clinically manifest through Branch A (persistence or rebound of low-level or missed preformed DSA), Branch B (memory B-cell reactivation with an anamnestic response), or both. The branches may coexist; Branch A has more direct clinical support, whereas Branch B remains predominantly mechanistic and inferential in HTW (16, 42–45).

DSA first detected after transplantation may represent previously unrecognized preformed antibody, rebound or re-emergence of historical DSA, a memory-mediated anamnestic response, or a primary response to the graft. Timing alone cannot distinguish these possibilities (9, 10, 39, 40, 42, 58–60).

Pregnancy was considered a plausible sensitizing source only when donor specificity corresponded to paternal HLA/eplets inherited by shared offspring and competing sensitizing events were considered. When offspring HLA/eplet data, paternity information, historical sera, or same-specificity continuity were unavailable, pregnancy-source attribution remained uncertain (10, 16, 24, 45, 58).

### 4.5 Effector injury: from DSA binding to early ABMR

Once donor-specific antibody binds graft endothelium, complement-dependent and Fc receptor-mediated mechanisms may contribute to microvascular inflammation and antibody-mediated rejection (41, 56, 61–64, 68–70, 73, 74). ABMR may be C4d-positive or C4d-negative. Banff criteria define the downstream pathologic phenotypes, but neither MVI nor ABMR is specific for pregnancy-shaped injury or can retrospectively establish pregnancy as the sensitizing source (65–67). DSA-negative or C4d-negative MVI may reflect non-HLA or nonhumoral mechanisms and therefore cannot be assigned to the proposed pathway without additional evidence (71, 72).

### 4.6 Integrated interpretation

Evidence is segmental and asymmetric: pregnancy-related paternal-antigen exposure and the downstream DSA–MVI/ABMR relationship are comparatively well supported (6, 15, 65–67), whereas donor-specific continuity from pregnancy exposure to pretransplant donor-reactive memory and same-specificity posttransplant recall remains unproven in HTW (10, 12, 13, 16, 28, 43, 50). **Table 2** summarizes the upstream exposure, latent reservoir, two proximal humoral branches, downstream injury, and key unverified links. The framework is supported by convergent evidence across these segments rather than by a fully traced continuous causal chain in a single prospective HTW cohort.

## 5 Determinants of axis expression and observed heterogeneity

**Figure 2:**
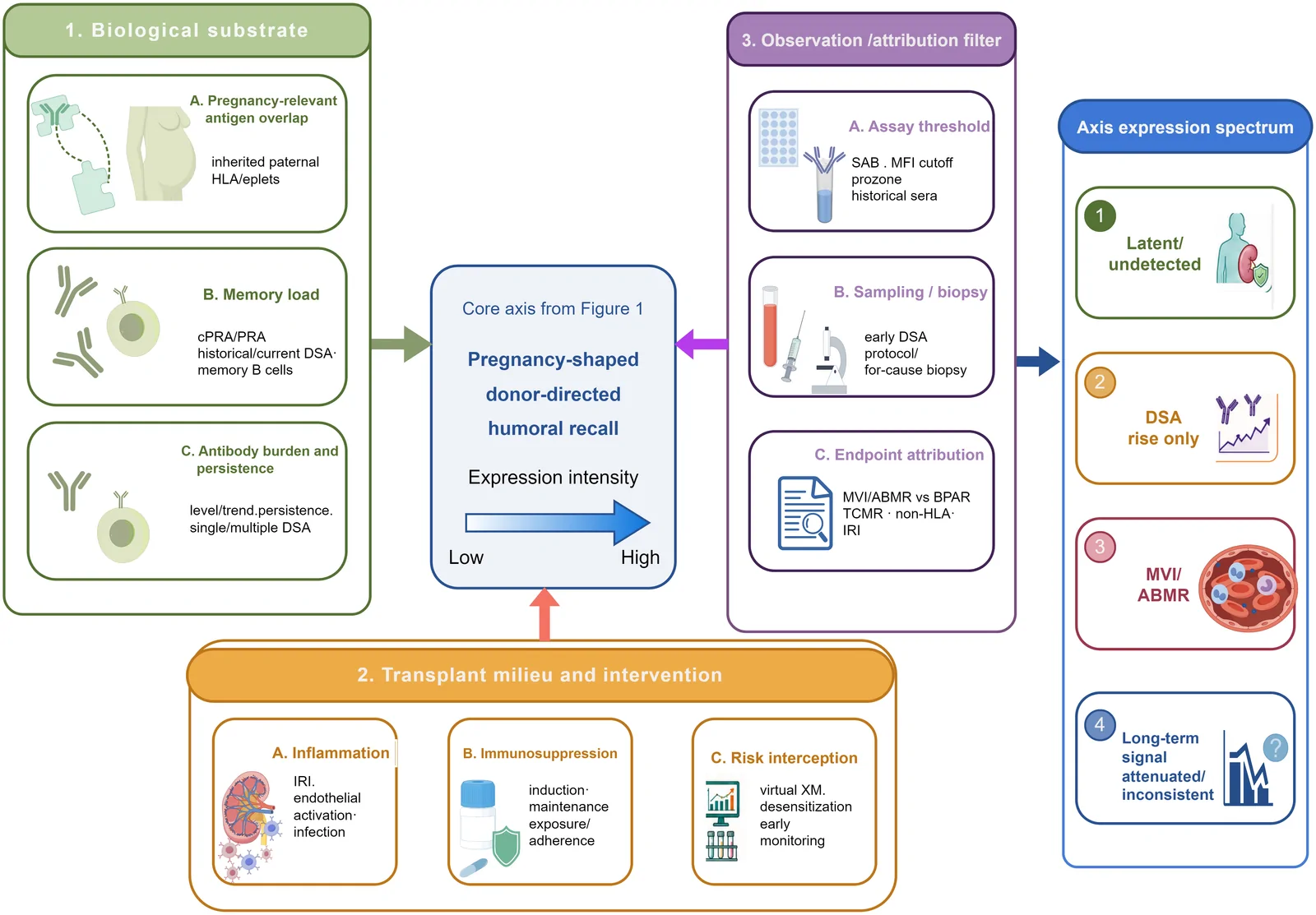
Determinants of heterogeneous expression and detection of the humoral recall axis. Expression of the core axis in **Figure 1** is shaped by biological substrate (pregnancy-relevant antigen overlap, humoral memory load, and antibody burden and persistence), transplant milieu and intervention, and observation/attribution factors (assay performance, sampling or biopsy strategy, and endpoint assignment). These domains influence whether donor reactivity remains latent, appears as an isolated DSA rise, progresses to MVI/ABMR, or produces attenuated and inconsistent long-term signals. The figure addresses heterogeneity in expression and detection rather than a separate mechanism. Selection, reporting, and competing-mechanism effects are discussed in Sections 5 and 7 and are not fully depicted.

The proposed pathway may manifest strongly or weakly or remain clinically silent. **Figure 2** groups the factors that shape whether the proposed pathway is expressed or detected into three domains: biological substrate, the transplant milieu and clinical intervention, and factors affecting observation and source attribution. These factors influence whether the axis remains latent, appears only as a DSA rise, progresses to MVI/ABMR, or is followed by an attenuated or inconsistent long-term signal. **Figure 2** summarizes variation in expression and detection; it does not replace the core mechanism in **Figure 1**.

Biological substrate includes overlap between paternal antigens inherited by shared offspring and the husband’s graft, current or historical DSA, PRA/cPRA, FCXM findings, donor-reactive memory B cells, and competing sensitizing events such as transfusion, other pregnancies, or prior transplantation (7, 16, 24, 42).

The transplant milieu and clinical management may modify or intercept pathway expression through ischemia–reperfusion injury, infection or inflammation, immunosuppressant exposure and adherence, risk-directed therapy, early DSA monitoring, and treatment. Contemporary SAB testing and virtual crossmatch primarily influence donor selection and risk interception rather than the underlying biological mechanism (10, 75, 76).

The observation and attribution filter includes assay sensitivity and thresholds, availability of historical sera and offspring/donor HLA data, timing of DSA sampling and biopsy, endpoint definition, and selection or reporting effects (10, 18, 20, 65, 66, 77, 78). These features influence whether MVI/ABMR is detected and whether a donor-specific response can be plausibly linked to shared pregnancy.

## 6 Clinical translation and incremental explanatory value

Routine risk assessment should remain based on cPRA, current and historical DSA, SAB testing, CDC-XM/FCXM, virtual crossmatch, and a complete history of sensitizing events (39, 40, 77–79).

Shared-pregnancy history, offspring HLA/eplet data, historical sera, and donor-reactive memory B-cell testing currently serve as supplementary interpretive variables or research-level measures rather than validated clinical thresholds (7, 16, 24, 42, 44). Accordingly, routine offspring HLA/eplet typing or paternity testing cannot currently be recommended as part of standard HTW evaluation. When such information is collected for a clinically justified purpose or within ethically approved research, appropriate informed consent and careful consideration of genetic privacy and the implications of paternity information are required (80).

Nor should shared pregnancy alone justify exclusion of a husband donor, routine desensitization, or intensified treatment (11–13). Additional variables would have clinical value only if they reproducibly improved identification of early DSA, MVI, or ABMR beyond established risk measures (10, 18, 20, 39, 40, 47, 77, 78, 81, 82).

## 7 Limitations and boundaries of inference

This mechanism-driven narrative review was based on a structured search, not a systematic review or meta-analysis (83). We did not conduct a formal systematic-review selection process, independent dual screening, study-level risk-of-bias assessment, or GRADE assessment of certainty (84). Evidence strength was therefore evaluated descriptively by clinical relevance and mechanistic node. Most HTW studies are small, single-center, and retrospective, with substantial variation in antibody testing, immunosuppression, rejection definitions, biopsy strategy, and follow-up (7). These differences limit direct comparison and may obscure modest long-term outcome effects.

The main gaps are incomplete pregnancy-source information and the absence of longitudinal evidence linking the proposed steps. Most studies do not jointly confirm shared pregnancy and biological paternity, map offspring HLA/eplets, analyze historical sera, or test pretransplant donor HLA-reactive memory B cells. Pregnancy can induce paternal HLA alloimmunization (15), and pregnancy-related memory B-cell studies support latent humoral memory after serum antibody wanes (16). Female-spousal studies show that early DSA may reflect missed preformed DSA (10), while Banff criteria define MVI and ABMR phenotypes (65, 66). No prospective HTW cohort has linked pregnancy exposure, pretransplant immune state, early posttransplant DSA kinetics, and Banff pathology across the entire sequence. Existing studies also rarely distinguish missed preformed DSA rebound from a memory B-cell–mediated anamnestic DSA response. Concordance across separate evidence segments does not prove a continuous causal chain.

Rapid posttransplant DSA or early rejection may also reflect transfusion, prior transplantation, other pregnancies, high overall sensitization, ABO or HLA incompatibility, inadequate immunosuppression, infection, or ischemia–reperfusion injury. DSA-negative or C4d-negative MVI may reflect non-HLA or nonhumoral mechanisms and therefore cannot be assigned to the proposed pregnancy-shaped humoral pathway without additional evidence (66, 67, 71, 72, 85). Exclusion of high-risk combinations, paired exchange, desensitization, case selection, and publication bias can attenuate or amplify reported associations. The proposed axis remains a hypothesis-generating framework, not a validated causal pathway or clinical decision instrument.

## 8 Conclusions and future directions

Shared pregnancy can constitute prior antigen exposure related to the current husband donor, but it does not make all HTW procedures uniformly high risk. Available clinical signals are concentrated among recipients with conventional humoral risk features, and MVI/ABMR remains a downstream phenotype rather than proof of pregnancy source (7, 10–13, 24, 65–67). Current evidence does not support donor exclusion or treatment changes solely because of shared pregnancy. The proposed two-branch axis is hypothesis-generating and requires prospective validation.

Prospective studies should integrate shared-pregnancy and paternity information, paternal HLA/eplets actually inherited by offspring, historical sera, pretransplant donor-reactive memory B-cell assays, serial DSA measurements early after transplantation, and contemporaneous Banff-classified biopsy findings, while testing incremental value beyond cPRA, current or historical DSA, and crossmatch results (16, 24, 67). Such studies should distinguish previously unrecognized or rebound preformed antibody, memory-mediated anamnestic responses, and primary responses to the graft.

## Supplementary material

**Supplementary Table S1** summarizes established terminology and interpretive cautions relevant to this review. **Supplementary Table S2** provides the complete PubMed/MEDLINE search strategies and retrieval counts and describes the role of targeted supplementary checks in Embase and the Web of Science Core Collection.
