# Supplementary material for: Pregnancy-shaped donor-directed alloimmune recall in husband-to-wife kidney transplantation: an integrative mechanistic framework
Source: Front Immunol. 2026 Sep 3;17:1936063. doi: 10.3389/fimmu.2026.1936063 (PMC13581896; doi:10.3389/fimmu.2026.1936063)
Supplement: Supplementary file 1 [file Table1.docx]

**Supplementary Table S1. Established terminology and interpretive cautions relevant to this review**

The entries below retain established transplant-immunology terminology and state the interpretive limits applied in this review.

| **Term** | **Established meaning used in this review** | **Interpretive caution** |
| --- | --- | --- |
| **Donor-specific antibody (DSA)** | An antibody directed against one or more HLA antigens expressed by the current kidney donor. | The term identifies donor specificity, not the sensitizing event. Pregnancy-source attribution must be assessed separately. |
| **Current pretransplant DSA** | DSA detected in the current pretransplant specimen using the assay and threshold applied during evaluation. | Distinct from historical DSA and from preformed DSA demonstrated only retrospectively or with more sensitive testing. |
| **Historical DSA** | A previous serum record of antibody directed against HLA expressed by the current donor, whether or not it remains detectable at transplantation. | Documents prior sensitization; it is not an active effector-cell compartment and is not itself a recall response. |
| **Low-level or missed preformed DSA** | DSA present before transplantation but detected at a low signal level or identified only retrospectively using stored serum, more sensitive testing, dilution, or interference control. | Must be demonstrated in a pretransplant specimen and should not be inferred solely from early postoperative detection. |
| **Persistent or rebound DSA** | Persistence after transplantation, or a postoperative rise, of the same specificity as prospectively detected, historical, or retrospectively demonstrated preformed DSA. | Timing alone does not establish persistence or rebound; pretransplant or historical evidence of the same specificity is required. |
| **Anamnestic antibody response** | An accelerated donor-specific antibody response after antigen re-exposure that is biologically compatible with reactivation of donor-reactive memory B cells. | Missed preformed DSA should be excluded and same-specificity continuity should be sought; early timing alone is insufficient. |
| **Donor-reactive memory B cells** | Memory B cells capable of recognizing donor HLA or producing donor-specific antibody after ex vivo stimulation in a specified research assay. | A research-level cellular finding. Detection does not by itself establish pregnancy origin or predict ABMR, and no assay or threshold has been validated for clinical risk assessment in HTW. |

Note. Pregnancy was considered a plausible sensitizing source only when donor specificity corresponded to paternal HLA/eplets inherited by shared offspring and competing sensitizing events were considered. When offspring HLA/eplet data, paternity information, historical sera, or same-specificity continuity were unavailable, pregnancy-source attribution remained uncertain. No new diagnostic categories or consensus terminology are proposed.

Abbreviations. ABMR, antibody-mediated rejection; DSA, donor-specific antibody; HLA, human leukocyte antigen; HTW, husband-to-wife kidney transplantation.
