# Supplementary material for: Pregnancy-shaped donor-directed alloimmune recall in husband-to-wife kidney transplantation: an integrative mechanistic framework
Source: Front Immunol. 2026 Sep 3;17:1936063. doi: 10.3389/fimmu.2026.1936063 (PMC13581896; doi:10.3389/fimmu.2026.1936063)
Supplement: Supplementary file 2 [file Table2.docx]

**Supplementary Table S2. PubMed/MEDLINE search strategies, retrieval counts, and supplementary database checks**

PubMed/MEDLINE provided the reproducible structured searches and record counts. Targeted supplementary checks in Embase and the Web of Science Core Collection, reference-list screening, and forward/backward citation tracking were used to broaden coverage.

| **Database/platform** | **Search date** | **Coverage** | **Evidence domain** | **Complete search strategy** | **Limits** | **Records retrieved** |
| --- | --- | --- | --- | --- | --- | --- |
| PubMed/MEDLINE (NCBI PubMed) | 3 July 2026 | Database inception–3 July 2026 | P1. High-sensitivity HTW/spousal kidney-transplant clinical search | (("Kidney Transplantation"[Mesh] OR "kidney transplant*"[tiab] OR "renal transplant*"[tiab]) AND ("husband-to-wife"[tiab] OR "husband to wife"[tiab] OR "husband wife"[tiab] OR H2W[tiab] OR HTW[tiab] OR spousal[tiab] OR spouse[tiab] OR "spouse donor*"[tiab] OR "spousal donor*"[tiab] OR "spousal kidney"[tiab] OR "living unrelated donor*"[tiab])) | No language or study-design filter; Entrez Date ≤ 3 July 2026. | 432 |
| PubMed/MEDLINE (NCBI PubMed) | 3 July 2026 | Database inception–3 July 2026 | P2. HTW/spousal kidney transplantation plus rejection/outcomes | (("husband-to-wife"[tiab] OR "husband to wife"[tiab] OR H2W[tiab] OR HTW[tiab] OR spousal[tiab] OR "spousal donor*"[tiab] OR "spousal kidney"[tiab]) AND ("kidney transplant*"[tiab] OR "renal transplant*"[tiab]) AND (rejection[tiab] OR "antibody-mediated rejection"[tiab] OR ABMR[tiab] OR BPAR[tiab] OR "graft survival"[tiab] OR "graft loss"[tiab] OR DSA[tiab] OR "donor-specific antibod*"[tiab])) | No language or study-design filter; Entrez Date ≤ 3 July 2026. | 76 |
| PubMed/MEDLINE (NCBI PubMed) | 3 July 2026 | Database inception–3 July 2026 | P3. Pregnancy/paternal antigen plus kidney-transplant clinical signal | (("Kidney Transplantation"[Mesh] OR "kidney transplant*"[tiab] OR "renal transplant*"[tiab]) AND (pregnan*[tiab] OR parity[tiab] OR parous[tiab] OR multipar*[tiab] OR "previously pregnant"[tiab]) AND (husband[tiab] OR spouse[tiab] OR "paternal HLA"[tiab] OR "paternal antigen*"[tiab] OR "inherited paternal antigen*"[tiab] OR "shared paternal"[tiab]) AND (rejection[tiab] OR "graft loss"[tiab] OR "donor-specific antibod*"[tiab] OR DSA[tiab] OR "antibody-mediated"[tiab])) | No language or study-design filter; Entrez Date ≤ 3 July 2026. | 26 |
| PubMed/MEDLINE (NCBI PubMed) | 3 July 2026 | Database inception–3 July 2026 | P4. Early ABMR/accelerated rejection with a spousal or pregnancy signal | (("antibody-mediated rejection"[tiab] OR ABMR[tiab] OR "humoral rejection"[tiab] OR "accelerated rejection"[tiab] OR "hyperacute rejection"[tiab] OR "early rejection"[tiab]) AND ("husband-to-wife"[tiab] OR "husband to wife"[tiab] OR spousal[tiab] OR spouse[tiab] OR pregnan*[tiab] OR multipar*[tiab]) AND ("kidney transplant*"[tiab] OR "renal transplant*"[tiab])) | No language or study-design filter; Entrez Date ≤ 3 July 2026. | 62 |
| PubMed/MEDLINE (NCBI PubMed) | 3 July 2026 | Database inception–3 July 2026 | P5. Shared paternal antigen/offspring-to-mother signal | (("offspring-to-mother"[tiab] OR "mother recipient"[tiab] OR "shared paternal"[tiab] OR "paternal antigen*"[tiab] OR "inherited paternal antigen*"[tiab] OR "anamnestic response"[tiab]) AND ("kidney transplant*"[tiab] OR "renal transplant*"[tiab]) AND (rejection[tiab] OR "graft loss"[tiab] OR antibody[tiab] OR DSA[tiab])) | No language or study-design filter; Entrez Date ≤ 3 July 2026. | 12 |
| PubMed/MEDLINE (NCBI PubMed) | 3 July 2026 | Database inception–3 July 2026 | P6. Exact-phrase sweep | "husband-to-wife kidney transplantation" OR "husband to wife kidney transplantation" OR "husband-to-wife renal transplantation" OR "husband to wife renal transplantation" OR "husband-to-wife renal transplants" OR "spousal donor kidney transplantation" | No language or study-design filter; Entrez Date ≤ 3 July 2026. | 312 |

Note. The reproducible structured search and retrieval counts were based on PubMed/MEDLINE. The six searches yielded 920 records before deduplication across searches; 680 unique PubMed records remained after removal of 240 duplicates. Targeted checks in Embase and the Web of Science Core Collection were supplementary and were not treated as independent systematic database searches; therefore, no separate database-specific strategies or retrieval counts are reported.

Abbreviations. ABMR, antibody-mediated rejection; BPAR, biopsy-proven acute rejection; DSA, donor-specific antibody; H2W/HTW, husband-to-wife; HLA, human leukocyte antigen; MeSH, Medical Subject Headings; tiab, title/abstract.
